# Editorial: IL-1 Inhibition

**DOI:** 10.3389/fphar.2019.00087

**Published:** 2019-02-12

**Authors:** Francesca Oliviero, Paolo Sfriso, Leonardo Punzi, Jean-Michel Dayer

**Affiliations:** ^1^Rheumatology Unit, Department of Medicine - DIMED, University of Padua, Padua, Italy; ^2^Faculty of Medicine, University of Geneva, Geneva, Switzerland

**Keywords:** IL-1 (interleukin-1), IL-1 blockade, anakinra, canakinumab, rheumatic diseases, inflammatory chronic diseases, autoinflammatory diseases

Interleukin 1 (IL-1) is a crucial mediator of the inflammatory response, playing an important part in the body's natural responses and the development of acute pathological conditions leading to chronic inflammation and tissue destruction. In the last decades, specific IL-1-targeting therapies have produced beneficial effects in a wide spectrum of IL-1 driven diseases, the most dramatic one being autoinflammatory disorders. Indeed, IL-1 continues to be a key attractive therapeutic target for many inflammatory, metabolic, skin, and heart disease. The main purpose of this Research Topic is to discuss and evaluate some current knowledge on IL-1 biology and the rational approach toward its blockade in different conditions. The topic covers both basic scientific as well as clinical aspects and includes 8 reviews, 8 original articles, 1 perspective, and 1 opinion. The topic focusing mainly on IL-1ß and its antagonism does not preclude for the importance of other members of the IL-1 family.

Back to history, Dayer et al. reminds the discovery of the monocytic origin of IL-1 in Rheumatology, the seminal description of the mechanism of IL-1 receptor antagonist (IL-1Ra) acting as ligand binding to its IL-1 receptor and its change levels in serum of adult-onset Still's disease.

The inflammasome, the expansion of the IL-1 family, as well as the role on the IL-1 and IL-1Ra in crystal induced arthropathies, osteoarthritis and in cartilage and bone biology, are discussed.

Following a useful update on IL-1, Cavalli and Dinarello review the importance of IL-1 blockade by anakinra, the recombinant form of the naturally occurring IL-1Ra. The Authors focus on anakinra treatment of a broad spectrum of acute as well as chronic inflammatory diseases, ranging from rare autoinflammatory diseases to common conditions such as gout and rheumatoid arthritis, type 2 diabetes, atherosclerosis, and acute myocardial infarction.

The efficacy of IL-1 blockade in autoinflammatory-associated skin diseases is extensively described in the article by Fenini et al. The Authors review the different IL-1ß antagonists available in the market, inflammasome inhibitors and IL-1α and IL-18 blockers under development and investigation in both monogenic and polygenic autoinflammatory diseases (Note of Editors: The biotherapies in adult-onset Still's disease and other rare inflammatory disorders, including blockade of IL-18 has been more recently discussed in the editorial of Guilpain et al., 2018).

The use of IL-1 inhibitors in the management of systemic juvenile idiopathic arthritis is discussed in the article of Giancane et al. As not all patients respond to anti-IL-1 therapy in that disease, the Authors argue about the possible causes of the different clinical responses focusing on the heterogeneous nature of systemic juvenile idiopathic arthritis.

Dusser and Koné-Paut discuss the key role of IL-1 inhibition in Kawasaki disease which shares phenotypical and immunological similarities with systemic juvenile idiopathic arthritis. Few clinical trials on IL-1 blockade in young patients affected with Kawasaki disease have been conducted and the Authors discuss the importance of this therapeutic approach to treat refractory forms.

The article of Colafrancesco et al. reports clinical results obtained from a large Italian multicentric retrospective observational study conducted in patients with adult-onset Still's disease. In this study, the efficacy and safety of two IL-1 inhibitors are evaluated in patient's refractory to other therapies. A good response was noted at 3 months after therapy onset in both groups.

Peiró et al. summarize the main experimental and clinical findings obtained with pharmacological IL-1β inhibitors in patients with diabetes mellitus and cardiovascular complications and discuss the perspectives of IL-1β inhibitors as novel therapeutic tools for treating these patients. (Note of Editors: In fact, just recently a large clinical study “CANTOS” shows promising results both in biological parameters and clinical outcomes (Ridker et al., 2017). At the 2018 Annual Meeting of the American College of Rheumatology, IL-1β inhibition with Canakinumab (Schieker et al., 2018) was shown to be associates with reduced rates of total hip and knee replacement and osteoarthritis).

Vitale et al. describe a nationwide Italian observational study on the on-label and off-label use of IL-1 inhibitors in children and adults. The Authors report demographic, clinical, and therapeutic data highlighting the wide use of these drugs as off-label indications in Italy. Their data confirm the good safety profile of IL-1 inhibitors.

The safety of anakinra combined with immunosuppressive drugs is discussed in the article of Mulders-Manders et al. They report three case series of patients undergoing renal transplantation treated with IL-1Ra in addition to common therapy during the pre- and post-operative period. These anecdotal reports illustrate a possible protective effect of anti-IL-1 therapy after solid organ transplantation. Peri- and postoperative use of anakinra is safe and effective in patients undergoing renal transplantation.

In the article of Assier et al. anti-cytokine vaccination approaches in autoimmune models are discussed with regards to IL-1 target. The Authors review the advantages and the limits of more specific targeting of IL-1β obtained with vaccination pointing out the promising results achieved with a peptide-based vaccine.

A second group of papers of this Research Topic focuses on basic scientific aspects. Novel insights in IL-1 production and release pathways, as well as its regulation in some rheumatic diseases are discussed.

Giuliani et al. review the role of P2X7 receptor (P2X7R), one of the molecules involved in IL-1β maturation. This ATP-gated ion channel acts through the recruitment of the NACHT-LRRPYD-containing protein-3 (NLRP) 3 inflammasome -caspase-1 complex. The authors summarize evidence supporting the role of the P2X7R in IL-1ß production, with special emphasis on the mechanism of release, a process that is still a matter of controversy. They discuss four different mechanisms of release: exocytosis via secretory lysosomes, microvesicles shedding from plasma membrane, release of exosomes, and passive efflux across a leaky plasma membrane during pyroptotic cell death.

In their article, Madej et al. investigate a novel mechanism of IL-1ß release and propose that innate memory appeared to limit the amount of active IL-1ß produced by macrophages in response to a bacterial challenge, while enhancing the responsiveness of monocytes.

The paper by Nasi et al. revisit the role of IL-1 in osteoarthritis. Through the meniscectomy model of murine osteoarthritis, the Authors do not support the role of IL-1α and ß as key mediators in that disease.

Armbruster et al. describe the protective effects of foamy viral vectors expressing IL-1Ra in an *in vitro* model of chondrogenesis. The Authors show that these vectors are capable of efficient gene transfer to mesenchymal stem cells which, in turn, efficiently block the effects of IL-1β. This gene transfer tool for mesenchymal stem cells could be a base for therapies for cartilage repair.

Oliviero and Scanu discuss various endogenous and exogenous factors involved in the self-limiting course of crystal-induced inflammation. The authors pay attention to novel mechanisms that intervene in the resolution of this process through the inhibition of IL-1ß production. Resolution of the inflammation remaining an important issue.

The importance of the regulation of IL-1ß production in crystal-induced inflammation is discussed in the paper of Khameneh et al. In their original research carried out in a murine model of urate crystal-induced peritonitis, the Authors identify the complement protein C5a as a key determinant of IL-1-mediated inflammatory cell recruitment. C5a generated upon MSU-induced complement activation increases neutrophil recruitment *in vivo* by promoting IL-1 production via the generation of reactive oxygen species, which activate the NLRP3inflammasome.

Finally, two articles investigate the beneficial effects of plant-derived natural compounds in *in vitro* and animal models of inflammation.

Liang et al. demonstrate that piperine can reduce adenosine triphosphate -induced pyroptosis in macrophages and to decrease systemic IL-1ß levels in a murine bacterial sepsis model.

The effect of curcumin is discussed in the original research by Kong et al. In this paper the authors show how this natural polyphenol effectively suppressed the expression of NLRP3 inflammasome in macrophages involving toll-like receptor (TLR)4/myeloid differentiation primary response 88 (MyD88)/nuclear factor kappa-light-chain-enhancer of activated B cells (NF-κB) and P2X7R pathways.

Overall, the various contributions provide an overview on therapeutic potential of IL-1 inhibition and discuss some original approaches on IL-1 production, regulation and action.

## Author Contributions

All authors listed have made a substantial, direct and intellectual contribution to the work, and approved it for publication.

### Conflict of Interest Statement

The authors declare that the research was conducted in the absence of any commercial or financial relationships that could be construed as a potential conflict of interest.
